# Supplementary material for: Implicit water model within the Zimm-Bragg approach to analyze experimental data for heat and cold denaturation of proteins
Source: Commun Chem. 2021 May 4;4:57. doi: 10.1038/s42004-021-00499-x (PMC9814862; doi:10.1038/s42004-021-00499-x)
Supplement: Supplementary file 1 — Supplementary Information [file 42004_2021_499_MOESM1_ESM.pdf]

Supplementary Info

Implicit water model within the Zimm-Bragg approach to analyze experimental data for heat and cold denaturation of proteins

Artem Badasyan,[1,*] Shushanik Tonoyan,[2] Matjaz Valant,[1,3] and Joze Grdadolnik[4]

[1]*Materials Research Laboratory, University of Nova Gorica, Vipavska 13, SI-5000 Nova Gorica, Slovenia*

[2]*Department of Molecular Physics, Yerevan State University, A. Manougian Str. 1, 0025, Yerevan, Armenia*

[3]*Institute of Fundamental and Frontier Sciences, University of Electronic Science and Technology of China, Chengdu 610054, China*

[4]*National Institute of Chemistry, Hajdrihova 19, SI-1000 Ljubljana, Slovenia*

(Dated: March 11, 2021)

* Corresponding author's email:abadasyan@gmail.com

CONTENTS

Supplementary Methods 2
Historical formulation of Zimm-Bragg model 2
Hamiltonian formulation of Zimm-Bragg model 4
Order parameter 6

Supplementary Discussion 7
Comparison of the fit with Go et al [10] 7

Supplementary References 7

References 7

SUPPLEMENTARY METHODS

Historical formulation of Zimm-Bragg model

Zimm and Bragg suggested a one dimensional model with short-range interactions, that sets rules for assigning statistical weights to repeat units of coil (c) and helical (h) conformations. They assumed that a particular state of a chain can be fully described by the states of all the oxygen atoms [1] with the help of a statement as to whether or not each is bonded to the hydrogen of the third preceding repeat unit. At this level of coarse-graining, the conformation of a chain comprised of N repeat units can be described by a sequence of $N - 3$ symbols, each having one of two values. The digit 1 represents a bonded oxygen atom state and the helical conformation and 0 stays for an unbonded atom and the coil conformation.

While there had been variants of the model [2], that considered dependence of statistical weights of a repeat unit from the states of a finite number of its neighbors, only a variant of Zimm-Bragg model with nearest-neighbor interactions survived. To estimate the partition function, Zimm and Bragg have suggested the following assumptions or rules:

(1) Every repeat unit can be found in either helical (H-bonded) or coil (unbonded) conformation;

(2) A statistical weight 1 is put into the correspondence to every repeat unit in coil conformation;

(3) A statistical weight of $s(T) = \exp\left[-\frac{\Delta G(T)}{T}\right]$ is put into the correspondence to every bonded repeat unit in helical conformation that follows another helical repeat unit. Here T stays for temperature, and $\Delta G(T) = G_{helix}(T) - G_{coil}(T)$ is the free energy (Gibbs or Helmholtz) cost of changing conformation from helix to coil per repeat unit;

(4) Every helical repeat unit that follows coil repeat unit, contributes a statistical weight of $s\sigma$.The cooperativity parameter $\sigma(= \text{const}) \ll 1$, by its definition, describes, how much is the original probability of helix growth, s, hindered by the fact that there is no preceding helical repeated unit; it is purely entropic by its nature, and can be interpereted as a cost for initiating helix in a sequence of coil repeat units.

On the basis of the rules above and following the transfer-matrix approach of Kramers and Wannier [3], the matrix of statistical weights $g(a,b)$ of the Zimm-Bragg model was suggested to be:

$$\widehat{M}_{Zimm-Bragg} = \begin{array}{|l|c|c|} \hline i \backslash i+1 & h & c \\ h & g(h,h) & g(h,c) \\ \hline c & g(c,h) & g(c,c) \\ \hline \end{array} = \begin{pmatrix} s & 1 \\ s\sigma & 1 \end{pmatrix} \tag{S1}$$

As usual, eigenvalue problem leads to the characteristic equation

$$\begin{gathered} |\widehat{M}_{Zimm-Bragg} - \widehat{I}\lambda| = \begin{vmatrix} s-\lambda & 1 \\ s\sigma & 1-\lambda \end{vmatrix} = (s-\lambda)(1-\lambda) - \sigma s = \\ \lambda^2 - (s+1)\lambda + s(1-\sigma) = 0, \end{gathered} \tag{S2}$$

which has two roots:

$$\lambda_{1,2}(s,\sigma) = \frac{1}{2}\left[1 + s \pm \sqrt{(1-s)^2 + 4\sigma s}\right]. \tag{S3}$$

The helicity degree in the thermodynamic limit reads

$$\begin{gathered} \theta_{Zimm-Bragg}(s,\sigma) = \frac{1}{N}\frac{\partial \ln Z}{\partial \ln s} = \frac{\partial \ln \lambda_1}{\partial \ln s} = \frac{s}{\lambda_1}\frac{\partial \lambda_1}{\partial s} = \\ \frac{s}{2\lambda_1}\left(1 + \frac{2\sigma - 1 + s}{\sqrt{(1-s)^2 + 4\sigma s}}\right). \end{gathered} \tag{S4}$$

Hamiltonian formulation of Zimm-Bragg model

In Ref. [6] we have suggested a microscopic formulation of the Zimm-Bragg model, using a Potts-like formulation akin to the more general, one-dimensional many-body model [7], simplified to the nearest neighbor level. Actually, all the results of this paper are a logical continuation and practical application of the solution of a more general theoretical problem in Ref. [7].

Assume $Q(\geq 2)$ possible values for the spin γ_i describing the state of the i-th repeated unit; $\gamma_i = 1$ value corresponding to values of the torsional angles ϕ_i and ψ_i from the helical region of the Ramachandran map, while the other $Q-1$ identical values correspond to torsional angles from the coil region. In brief, Q has the meaning of the phase space volume of our problem. The energy of interaction is assumed to be different from zero when both γ_i and γ_{i+1} are equal to 1. The corresponding spin Hamiltonian is,

$$-\beta H_{Zimm-Bragg}(\{\gamma_i\}) = J\sum_{i=1}^{N}\delta(\gamma_i,1)\delta(\gamma_{i+1},1), \tag{S5}$$

here δ's stand for the Kronecker symbols and $\beta = 1/\kappa_B T$ is the inverse thermal energy. We address the interested reader to Ref. [8] for detailed comparison of the Potts-like model defined by Eq. (S5) with the classical 1D Potts model.

The partition function Z can be obtained using the standard transfer matrix technique [3, 9]

$$Z_{Zimm-Bragg}(W,Q) = \sum_{\{\gamma_i\}}\prod_{i=1}^{N}(\mathbf{M})_{\gamma_i,\gamma_{i+1}}, \tag{S6}$$

where $(\mathbf{M})_{\gamma_i,\gamma_{i+1}}$ are the elements of the $Q\times Q$ matrix

$$\mathbf{M}(Q\times Q) = \begin{pmatrix} e^J & 1 & ... & 1 \\ 1 & 1 & ... & 1 \\ ... & ... & ... & ... \\ 1 & 1 & ... & 1 \end{pmatrix}. \tag{S7}$$

Since $\mathbf{M}(Q\times Q)$ from Eq. (S7) contains only two linearly independent rows or columns, the order of the non-trivial part of the characteristic equation is also equal to two. Indeed (see Ref. [6] for derivation), the eigenvalue problem $|\widehat{M} - \widehat{I}\Lambda| = 0$, with the help of elementary

transformations (which do not alter the determinant) reduces to

$$\Lambda^{Q-2} \times \det \begin{pmatrix} e^J - 1 - \Lambda & e^J - 1 \\ 1 & Q - \Lambda \end{pmatrix} = 0. \tag{S8}$$

Neglecting the $Q-2$ trivial eigenvalues results in the characteristic equation

$$\Lambda^2 - \Lambda(W - 1 + Q) + (W-1)(Q-1) = 0, \tag{S9}$$

where $W = e^J$, $J = U/T$, $U(>0)$ is energy and T is temperature. Resulting two roots are:

$$\Lambda_{1,2} = \frac{1}{2}\left[W - 1 + Q \pm \sqrt{(W - Q + 1)^2 + 4(Q-1)}\right]. \tag{S10}$$

Surprisingly, a simple change of variables

$$\lambda \to \Lambda/Q; \quad s \to (W-1)/Q; \quad \sigma \to 1/Q \tag{S11}$$

in Eq. (S8) yields

$$\det \begin{pmatrix} s - \lambda & s \\ \sigma & 1 - \lambda \end{pmatrix} = \lambda^2 - \lambda(s+1) + s(1-\sigma) = 0, \tag{S12}$$

which exactly coincides with the characteristic equation for the Zimm-Bragg model given in Eq. (S2). Therefore, the Hamiltonian in Eq. (S5) provides exactly the same thermodynamics of the Zimm-Bragg model and, hence, can be considered equivalent to it. Throughout the paper we derive formulas from the Hamiltonian formulation and will therefore widely use the $\{W, Q\}$ parametrization. Nevertheless, upon necessity we can convert the results to the original $\{s = \frac{W-1}{Q}, \sigma = \frac{1}{Q}\}$ parameters using Eq. (S11). Interesting enough, Eq. (S11) immediately reveals, that the parameters W and Q are independent from each other, while s and σ are not.

The partition function in Eq. (S6) can be simplified to read:

$$\begin{aligned} Z(W,Q) = C_1\Lambda_1^N + C_2\Lambda_2^N = \Lambda_1^N\left[C_1 + C_2\left(\frac{\Lambda_2}{\Lambda_1}\right)^N\right] = \\ \Lambda_1^N\left[C_1 + C_2 e^{-N/\xi}\right] \xrightarrow[N\to\infty]{} C_1\Lambda_1^N \approx \Lambda_1^N, \end{aligned} \tag{S13}$$

where $C_1 = \frac{Q-\Lambda_2}{\Lambda_1-\Lambda_2}$, $C_2 = \frac{\Lambda_1-Q}{\Lambda_1-\Lambda_2}$.

Order parameter

In the model defined by Eq. (S5), the H-bond formation between two peptide units takes place, when they both are in the same ordered conformation ($\gamma = 1$). The order parameter (helicity degree) can be defined as the average relative number of intramolecular H-bonds:

$$\theta(W,Q) = \frac{< \sum_{i=1}^{N} \delta(\gamma_i, 1)\delta(\gamma_{i+1}, 1) >}{N} = \frac{1}{N} \frac{\sum_{\{\gamma_i\}} e^{J \sum_{i=1}^{N} \delta_i^{(2)}} \sum_{i=1}^{N} \delta_i^{(2)}}{Z} = \frac{1}{N} \frac{\partial \ln Z}{\partial \ln W}. \tag{S14}$$

Using the map Eq. (S11), we can re-write the order parameter in Zimm-Bragg variables as

$$\theta(s,\sigma) = \frac{1}{N} \frac{\partial \ln Z}{\partial \ln s} \frac{\partial \ln s}{\partial \ln W} = \frac{1}{N} \frac{\partial \ln Z}{\partial \ln s} \frac{s+\sigma}{s} = \theta_{Zimm-Bragg}(s,\sigma) \frac{s+\sigma}{s}. \tag{S15}$$

It is defined through the Hamiltonian and differs from the classical formula Eq. (S4) by a factor of $\frac{s+\sigma}{s}$, which is very close to 1 for small σ. Because of the better reasoning behind, we are using Eq. (S15) throughout the paper as a default definition of order parameter.

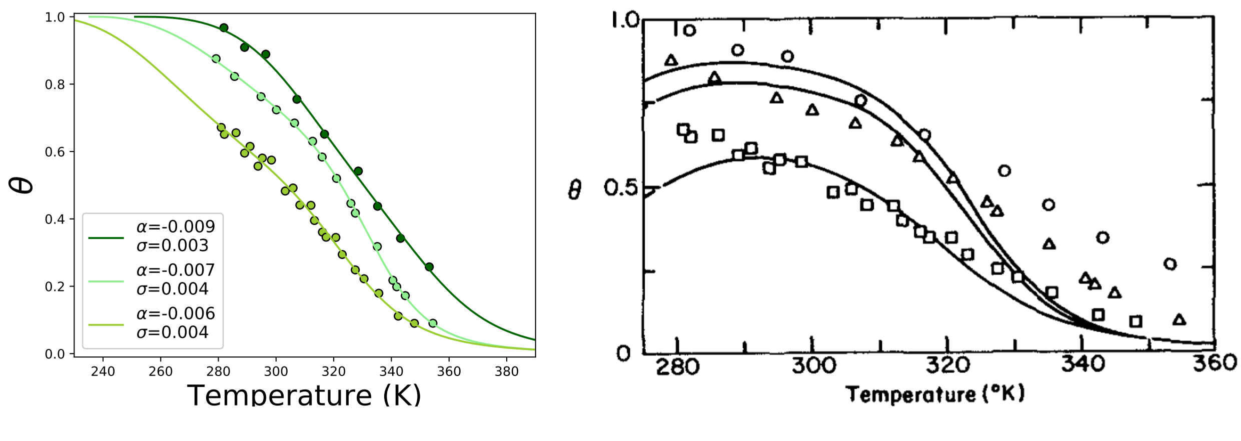

SUPPLEMENTARY FIGURE 1. **Comparison of fits.** Picture on the left is our result, and picture on the right is Figure 3, Reprinted from Go, M., Go, N. & Sheraga, H.A. Molecular Theory of the Helix–Coil Transition in Polyamino Acids. III. Evaluation and Analysis of s and σ for Polyglycine and Poly-l-alanine in Water. J. Chem. Phys. 54, 4489-4503 (1971), with the permission of AIP Publishing.

SUPPLEMENTARY DISCUSSION

Comparison of the fit with Go et al [10]

We have mentioned poor quality of fit performed in the past. To illustrate our point, we show it in Supplementary Figure 1 and one can obviously see, that the original fit fails even qualitatively, although a large (> 5) number of fitting parameters had been used by the authors. The experimental points indicate no evidence of cold denaturation, while the fitted curves clearly pinpoint the maximum. Our results, instead, perfectly agree with the data. As shown in Table 1 (see the main file), first three entries (corresponding to the data from Ref. [10]), have R^2 values larger that 0.99 in all three cases.

SUPPLEMENTARY REFERENCES

[1] Zimm, B. H. & Bragg, J. K. Theory of the Phase Transition between Helix and Random Coil in Polypeptide Chains. *J. Chem. Phys.* **31**, 526-535 (1958).

[2] Poland, D. & Scheraga, H.A. *Theory of helix-coil transitions in biopolymers* (Academic Press, New York, 1970).

[3] Kramers, H.A. & Wannier, G.H. Statistics of the two-dimensional ferromagnet. *Phys. Rev.* **60** 252-262 (1941).

[4] Landau, L. & Lifshits, E. *Statistical Physics* (Pergamon, Oxford, 1988).

[5] Cantor, C. & Schimmel, T. *Biophysical Chemistry* (Freeman and Co., San-Francisco, 1980).

[6] Badasyan, A. V., Giacometti, A., Mamasakhlisov, Y. Sh., Morozov, V. F. & Benight, A. S. Microscopic formulation of the Zimm-Bragg model for the helix-coil transition. *Phys. Rev. E* **81**, 021921:1-4 (2010).

[7] Ananikyan, N.S., Hajryan, Sh. A., Mamasakhlisov, E.Sh. & Morozov, V.F. Helix-coil transition in polypeptides: A microscopical approach, *Biopolymers* **30**, 357-367 (1990).

[8] Badasyan, A., Giacometti, A., Podgornik, R., Mamasakhlisov, Y. & Morozov, V. Helix-coil transition in terms of Potts-like spins. *Eur Phys J E*, **36**, 46:1-9 (2013).

[9] Flory, P. *Statistical Mechanics of Chain Molecules* (Interscience, New York, 1969).

[10] Go, M., Go, N. & Sheraga, H.A. Molecular Theory of the Helix–Coil Transition in Polyamino Acids. III. Evaluation and Analysis of s and σ for Polyglycine and Poly-l-alanine in Water. *J. Chem. Phys.* **54**, 4489-4503 (1971).
